# Study of temporal behaviour of meteorological drought using innovative polygon trend and scaling hypothesis methods in Kolasib district, Mizoram, India

**DOI:** 10.1038/s41598-025-00053-y

**Published:** 2025-06-02

**Authors:** Bivek Chakma, G S Yurembam, Deepak Jhajharia, G T Patle, Rakesh Salam, Saurav Saha

**Affiliations:** 1 College of Agricultural Engineering and Post Harvest Technology, Central Agricultural University, Imphal, India; 2 ICAR Research Complex for NEH Region, Sikkim Centre, Tadong, Gangtok, Sikkim India

**Keywords:** Meteorological drought, Long-term persistence, Scaling hypothesis, Innovative polygon trend analysis, Mizoram, Northeast India, Climate sciences, Hydrology

## Abstract

Drought, one of the most frequent water-related disasters, puts a great deal of strain on agriculture and associated activities in all types of climates. North-east India (NEI) having residual rainforests is also affected by this slowly unfolding disaster inspite of this region receiving the highest annual global rainfall. The multi-time scale Standardized Precipitation Index (SPI 1-month and 3-month) was used to determine the prevalence of meteorological drought in Kolasib (having humid subtropical climate, Cwa) site from Mizoram, a mountainous state situated in the southern tip of the NEI. To identify the nature of the drought, an innovative polygon trend analysis (IPTA) along with the change point detection (Pettitt and Pruned Exact Linear Time methods) and the long-term persistence (LTP) of meteorological drought under scaling theory was examined. The highest drought severity, drought duration and interval based on the SPI-1 (SPI-3) at Kolasib were observed as -11.4, 7 months from October, 1990 to April, 1991 and 176 months from March, 1996 to October, 2010 (-34.6, 17 months from December 1989 to April 1991 and 222 months from September, 1993 to February, 2012), respectively. Further, the IPTA analysis was carried out on the SPI-1 (SPI-3) month time series, and the monthly standard deviation obtained indicate that the highest decreasing trend transition occurred from May to June (July to August), i.e., trend slope of -9.45 (slope of -9.6) points of more and stronger drought event’s occurrence in crucial months of monsoon season. The PELT method observed the most drought conditions from June to August and November to January, which were almost similar to the results obtained through the IPTA. Trend analysis of drought events of both SPI-1 and SPI-3 through the LTP revealed that all twelve months observed long–term drought effect in Kolasib. The strongest LTP (Hurst estimate more than 0.90) for the SPI-3 were observed in June to September and November to January, respectively. However, no significant trends exist in the LTP, which indicated a longer drought cyclic pattern in Kolasib. It is evident from these findings that droughts in Kolasib are not only intensifying but also becoming more prolonged, necessitating proactive and science-based policy measures for drought mitigation in the region.

## Introduction

Drought is one of the natural climatic disasters characterized by a sustained shortage in rainfall over a region. It is also defined as a long-lasting regional phenomenon that deviates from the normal over a considerable geographic area and a prolonged length of time resulting in a momentarily large reduction in the availability of water^1^. Water resource management has become more challenging because of the negative consequences of drought on the economy, environment, public health, energy production, and water supply^2^. Drought occurs in all types of climates and is not limited to arid or semi-arid regions. An area with a dry climate is more likely than humid to experience moderate to severe drought more frequently. However, the frequency and intensity of drought may alter depending on the kind of climate. A study revealed that the North Eastern Region (NER) has seen an unprecedented increase in drought occurrences in the recent two decades when compared to the western part of India^3^. Additionally, they found that the probability of drought occurrence in the NE (Western) region of India was 54% (27%). They found that the drought associated with El Nino was not so strong in NE^3^. Also, the drought trend was expected to continue rising in both short-term and long-term observations over NE India, and showed a significant increase in the 1990 s and is projected to persist^4^.

The probabilistic characterization of drought is crucial for the accurate assessment of water resources and their planning and management, given the scarcity of water caused by meteorological drought in nearly all types of environments, as well as the widespread and well-established global warming in the changing climate scenario^5,6^. Traditionally, a number of indicators based on shortage in rainfall and discharge are used to assess the drought situation in a region^7^^9^. Around the world, several techniques have been used to categorise drought events. The Palmer Drought Severity Index (PDSI) is a tool that has been extensively used to identify a drought event by various authors^10,11^. These methods range from simple methods based on rainfall deviation from the mean rainfall to complex methods requiring various climatic parameters. The Standardized Precipitation Index (SPI), one of the leading indices for the evaluation of drought conditions in monitoring and warning systems, enables the multi-scale appraisal of drought phenomena^12^. The SPI is based on the likelihood of precipitation at any time frames. Due to its simplicity, spatial invariance, and probabilistic nature, the SPI has gained popularity and numerous scholars have used the SPI in their studies^13,14,15,16,17,18,19,20^. The SPI, a univariate drought index, shows substantial relevance in the analyses of temporal, spatial, and precipitation probability distribution, consequently aiding in the detection of drought across extensive geographic territories and multiple temporal frameworks^16,17^. The SPI has been used for drought monitoring in more than 70 countries and has demonstrated notable performance and dependability in a variety of settings^21,22,23^. The incorporation of SPI into regionaland local drought assessment frameworks demonstrates its effectiveness in identifying drought patterns and facilitating comparisons of drought occurrences across various temporal regimes^24^. However, the effectiveness of the SPI as a drought index can be influenced by external climatic factors^21^.

Monitoring meteorological drought is a fundamental and primary task for assessing various drought types, i.e., agricultural, hydrological, socio-economic, and groundwater drought — since rainfall deficits primarily drive all drought conditions. Advanced statistical analyses of climatic time series, particularly meteorological data, employ methodologies, such as the Mann-Kendall trend test (MKt) and Sen’s slope estimator to detect trends and quantify changes over time. For instance, a study analysing rainfall trends in Himachal Pradesh, India, utilized these methods to identify significant increases in pre-monsoon and post-monsoon rainfall, aiding in effective water resource management and agricultural planning^25^. The primary focus is temporal trends, while spatial trend studies outcomes are important for regional evaluations. Numerous researchers have focused on trend detection using conventional method of MKt^26,27^following the popular trend researchers^28,29,30,31^along with Theil-Sen slope, this approach aids in determining the trend’s magnitude^32^. In most cases, trend analysis is based on non-parametric approaches, including the MKt or Sen’s slope, which are more suitable than parametric methods for handling non-normally distributed datasets^33^. These analytical procedures illustrate distinct constraints linked to the null hypothesis (H_o_), which indicates the presence of serial correlation within the data set^34,35^. To overcome this specific challenge, the Innovative Trend Analysis (ITA) methodology was proposed by Şen (2012)^36^and subsequently employed in numerous research studies related to different durations of precipitation time series analysis globally^37,38,39,40,41^. In light of the extensive application of the ITA in recent times, several enhancements to this methodology have been suggested and the Innovative Polygon Trend Analysis (IPTA) is one such upgrade^42^. The IPTA was developed by expanding the ITA approach because these parametric and non-parametric approaches lack seasonal trend behaviour and periodic variation, which can be highly helpful for managing goals and activities^43^. The IPTA approach bridges the gap in trend analysis by identifying trends, determining trend transitions, and providing a trend polygon for improved numerical interpretation. In particular, to improve the ability to identify trends within a given dataset, the IPTA methodology makes it easier to identify trend transitions between consecutive segments of two equivalent portions that originate from the original hydro-meteorological time series and the result of which is a trend polygon^43^. The practicality of IPTA has also been investigated in many other studies^37,44 – 45^. The IPTA approach is thought to be an excellent, sensitive, and practical tool for quantitatively identifying trend analyses through length and trend slope as well as transactions from one period to another^46^. A parametric and non-parametric trend analysis for monthly change point detection in the time series is used to support the IPTA, these two methods will give more precious information about monthly drought conditions. Change point detection (CPD) identifies time steps when one model changes to a new model such as a change in the mean value. The Pettit’s test of rank based non-parametric test is the most commonly used method to detect single change point and has been used in monthly SPI timescales to observe the most significant single break^47^. The test is designed to detect only one significant change in the time series. If multiple shifts exist, the test may only capture the most dominant one, making it less suitable for complex datasets with multiple structural changes^48^. Additionally, the test has reduced power in detecting changes in extreme values, as it performs better when analysing median or central quintiles rather than maxima or minima. However, to overcome this limitation by Pettitt test, a new approach of CPD is the PELT (Pruned Exact Linear Time**)**test, which is a parametric method to detect maximum or multiple significant breaks in the time series^49^. In this study, both methods (Pettitt and PELT test) were used to observe the most significant break point for single and multiple CPD and to identify the best possible output of change point trend.

A district-wise investigation of rainfall trends in seasonal and annual timescales revealed a significant decline in total rainfall in Mizoram^31^. Despite having abundant water resources, the area has been experiencing water shortages in the past few decades in the state^50^. The state is experiencing severe consequences which significantly impact the traditional and tribal agricultural practices in mizoram^51^. There are relatively very few studies on drought analysis in the far north eastern state of Mizoram situated in close proximity to Bangladesh and Myanmar. In this study, the selected research area, i.e., Kolasib site has not been subjected to IPTA on the multi-time-SPI based drought characteristics, which motivated us to search the drought monitoring transition from one month to another (e.g., January to February) of each SPI month (SPI-1 and SPI-3). Further, employing the Scaling Hypothesis will give us the knowledge of short-term and long-term persistence of drought in the far north-eastern site. Since the MKt and other traditional trend tests depend on the assumption that observations are independent, which may not be the case with hydroclimatic data. Long-term persistence (LTP) is suggested by the Hurst-Kolmogorov (HK) dynamics, which means that previous values affect future values and affect the significance of statistical trends.

The goal of the study is to use the multi-time-scale SPI (SPI-1 and SPI-3) to determine the prevalence of meteorological drought in Kolasib, a site having humid subtropical dry winter climate and located in the state of Mizoram, NEI. Since the area depends on rainfall for numerous purposes, including industrial, agricultural, and the replenishment of its water resources (rivers, streams, etc.). A trend test using an IPTA is performed to track the monthly drought transition from one month to the next, the change point detection to observe the monthly trend, and the Scaling Hypothesis to track the long-term persistence of drought over the region. In light of this, the present study was carried with the following objectives are: To identify dry episodes as maximum drought characteristics of duration, severity and drought interval over Kolasib (Mizoram), NE region using the SPI; to identify the transition of drought from one month to the next using IPTA and change point detection using Pettitt and PELT method of every drought period; and to construct future drought forecasting by determining the long-term persistence of drought’s trend under scaling hypothesis. Understanding all this information will help with drought preparedness, mitigation, and adaptation in the NEI. Also, the study will help policymakers to create proactive, data-driven solutions. Governments and stakeholders may improve early warning systems, manage water resources more effectively, and create sustainable solutions to increase drought resilience by incorporating these findings into policy frameworks.

## Methods

### Study area and data acquisition

**Fig. 1:**
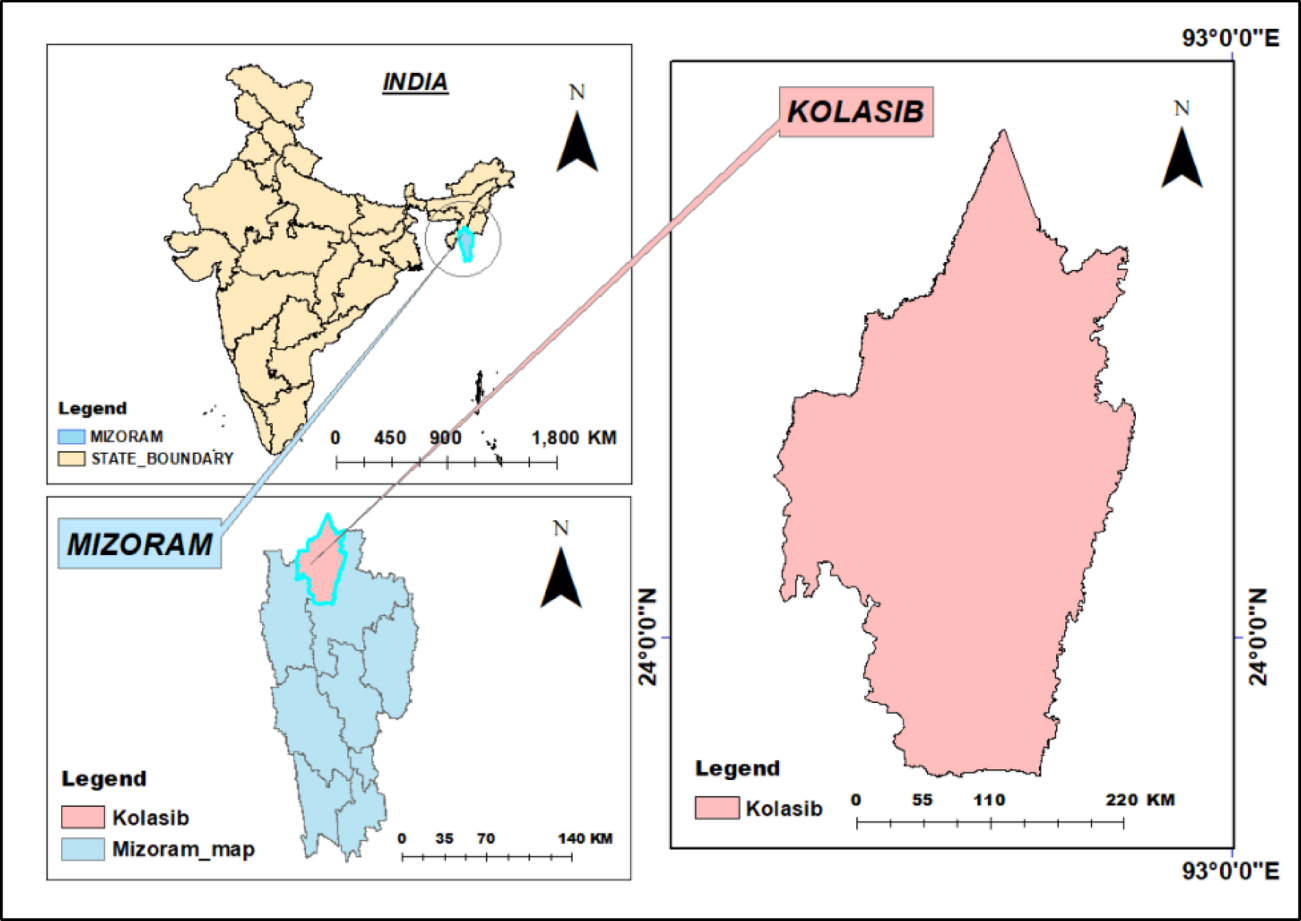
Map of the study area.

**Fig. 2:**
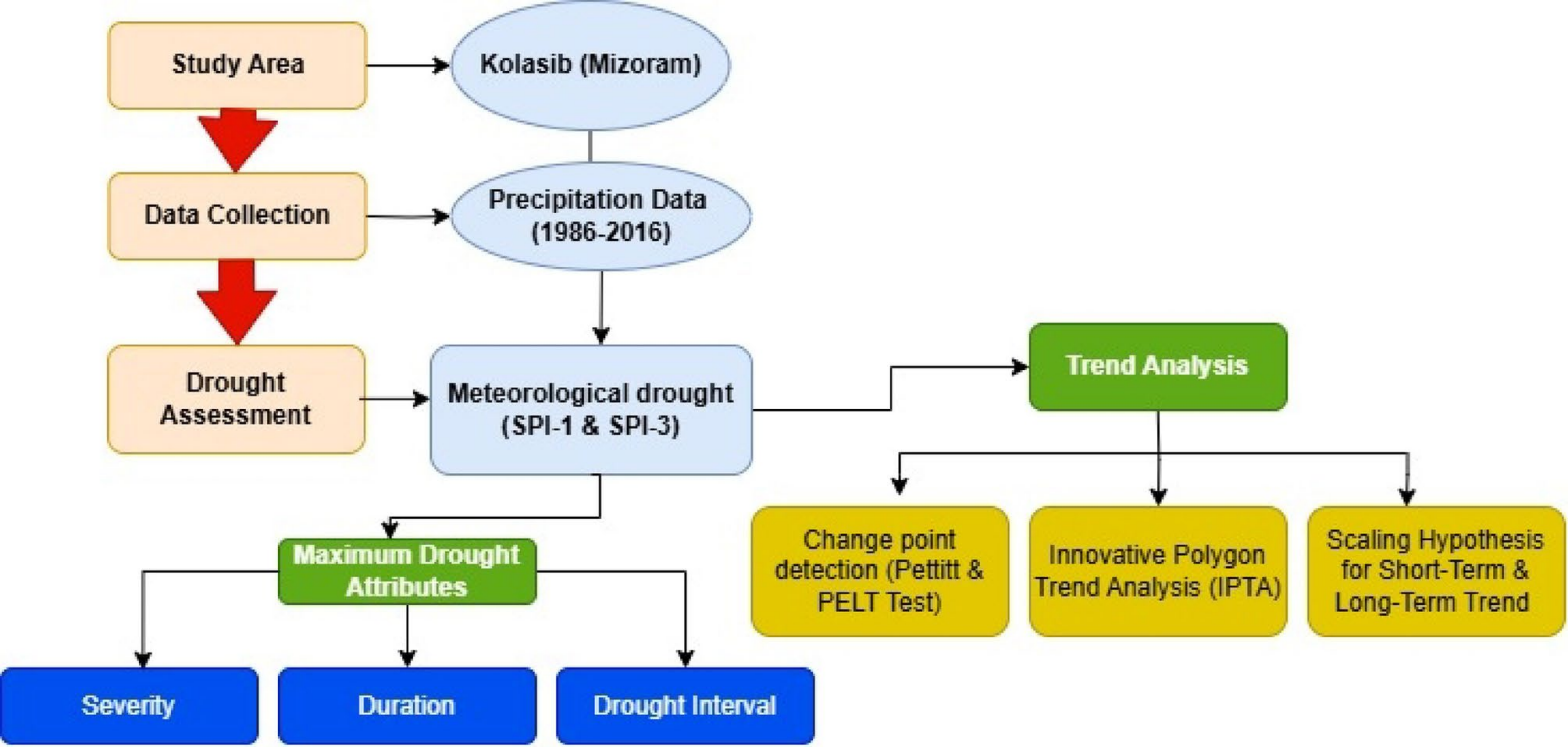
Flow chart of the study.

Northeast India (NEI), with its abundant biodiversity and monsoon-dominated climate, has two main river basin systems—The Brahmaputra and Barak Rivers—and approximately 60% of its land are covered in forests. Mizoram, one of the mountainous states of the NEI, is situated in the southern tip of the NE region, and it shares international borders with Bangladesh to the west, and Myanmar to the east and south, and domestic borders with the NE Indian states of Assam, Manipur, and Tripura. The archaeological evidence uncovered in the Vangchhia region supports the theory of early civilisations in Mizoram since around 600 BC. The Kolodyne River also called the Chhimtuipui River is the largest river (138.5 Km) in the state of Mizoram, India. This river originates from Myanmar and flows southwards into Mizoram. Another river called The Tlawng (Dhaleswari) river is the longest in Mizoram, which is about 185 km and it directly drains into the Barak River. The only river flowing through Kolasib is Serlui River (56 km. long). The region is home to one of the last remaining rainforests on the Indian subcontinent, and the majority of the cropland is rain-fed. In the present study, we selected Kolasib as our study area for identifying the drought events and studying their characterisation. Kolasib is selected because it is an important transit point connecting Mizoram with the rest of India. Kolasib (latitude 24.225°N and longitude 92.678° E and elevation of 888 m above mean sea level) is having humid subtropical climate, which is Cwa type climate as per the Köppen–Geiger climate classification. The geographical area of Kolasib district is 1473 sq km with average annual rainfall of 2703 mm and mean temperature of 24.74ºC. Kolasib is mostly dependent on indigenous agricultural practices, i.e., slash/burn). Investigation of a particular site with potential historical data and advanced statistical techniques can provide additional information about climate change and future trends for the NE state. The monthly rainfall data was collected from the Kolasib site for a period of 1986 to 2016 (31 years) from the regional centre of ICAR, Kolasib (Mizoram). The study area map is shown in Fig. 1 and the detailed graphical methodology or flowchart for investigating the drought events is presented in Fig. 2.

### Standardized precipitation index

The SPI is one of the most widely used drought indicators (DI) that has been used by several authors in different parts of the world^14,15,52,53^. It may be estimated on a range of timeframes, i.e. SPI-1, SPI-2, SPI-3, etc. (1-month, 2-month, 3-month, etc.), characterising various drought variables. The SPI is calculated using historical long-term precipitation data of Kolasib. In this study, the SPI values are approximated using the SPI-1 and SPI-3 for monthly time scales^12^. SPI-1 is used for short-term weather patterns and moisture conditions. It is beneficial for identifying short dry or rainy periods that impact soil moisture, agriculture and weather research, while SPI-3 represents changes in seasonal precipitation, which is helpful for hydrological and agricultural research, particularly in regions where seasonal rainfall is essential. The study area is dependent on seasonal rainfall for various activities. Therefore, employing both time scales (SPI-1 and SPI-3) will enhance more information about short term (monthly) and long-term (seasonal) drought.

The backward moving average of length (l) (or cumulative precipitation of the previous months up to the current month, say SPI-3 for January to March) estimated on month (m) may be computed as follows to evaluate SPI using monthly total rainfall (say x) for a selected time (T);

1 $${\delta _m} = \;\frac{{{x_m} + \;{x_{m - 1}} + \ldots + {x_{m - l\; + 1}}\;\;\;}}{l}$$

Since, its assumes a stationary probability distribution, meaning its parameters remain constant over time^54^. Then the probability distribution function (pdf) is fitted to Gamma distribution^55^ which is given as,

2 $$f\left( {x|\;\alpha ,\;\beta } \right) = \frac{1}{{\Gamma (\alpha ){\beta ^\alpha }}}{x^{\alpha - 1}}{e^{ - \beta /x}},\;\;\;\;\;\;\;\;where,\;x> 0,\;\alpha> 0,\;\beta> 0$$

Where,$\:\:\alpha\:,\:\beta\:\:and\:{\Gamma\:}\:\left(\:.\right)$ are the shape parameter, scale parameter and gamma function. Once the pdf is observed, the SPI ($\:{{\Delta\:}}_{m})$is perform the transformation into standard normal distribution by using inverse cumulative distribution function (cdf), which give the SPI^12^, it is given as;

3 $$\:{{{\Delta\:}}_{m}=\:\varPhi\:}^{-1}\left(F\left({\delta\:}_{m}\right)\right)$$

Based on the SPI values the drought classification are categorised into following range as shown in Table 1.

**Table 1:** Standard SPI values of drought classification (Sharma et al., 2021).

Drought Characterization	SPI values
Moderate	− 1.00 to − 1.49
Severe	− 1.50 to − 1.99
Extreme	− 2.00 and less

### Change point detection

Change Point Detection (CPD) embodies a statistical technique applied to determine moments in a temporal sequence or time series where the essential process or distribution experiences considerable changes. For the analysis of time series data in a variety of fields, change point detection is crucial. It facilitates decision-making, anomaly detection, forecasting model improvement, and structural break identification. For single point detection techniques, such as Pettitt’s test can be used using a rank-based approach^47^and PELT a parametric test for multiple change detection is done using an optimization-based cost function^49^. Discussion of the two methods is given below:

### Pettit test

A nonparametric, rank-based statistical technique for locating change points in a data series is the Pettitt test^47^. It has the capability to detect the change year (shifting) in the time series and identify the most significant single change point in the middle of the series. It takes into account a series of random variables X _1_, X _2_, …., X_T_, where a possible change point at time “$\:\tau\:$” splits the series into two parts with different distribution functions. The built up of mathematical functions derived from^48,56^ are given below:

4 $${F_{1\;}}({X_t}),\;\;t = 1,\;2,\;3,\; \ldots \ldots .,\;\tau ,\;and\;{F_{2\;}}({X_t}),\;\;t = n + 1,\; \ldots \ldots .,\;T\;$$

To detect the change point, the hypothesis can be tested as follows; $$\:\left\{\begin{array}{c}{F}_{1}\left(X\right)={F}_{2}\left(X\right);{H}_{0}\left(no\:change\:point\right)\\\:{F}_{1}\left(X\right)\ne\:{F}_{2}\left(X\right);{H}_{1}\left(Change\:point\:detect\right)\end{array}\right.$$

To observed the maximum absolute value of the U statistic (U is a vector of test statistics computed for each possible change point), which represents the test statistic $\:\left({U}_{t,\:T\:}\right)\:\:$, the Mann-Whitney^57^for two sample (before and after change point) is given by^47^:

5 $${U_{t,\;T\;}} = \sum\limits_{i = 1}^t {\sum\limits_{j = t + 1}^T {sgn(} } {X_i} - {X_j}),\;1\; \leqslant t < T\;\;$$

Where, sgn (.) is the sign function:

$$\:sgn\:\left(x\right)=\left\{\begin{array}{c}1\:\:\:\:\:,if\:x>0\\\:0\:\:\:\:\:,\:if\:x=0\\\:-1\:\:\:,\:\:if\:x<0\end{array}\:\:\:\:\:\:\:\:\:\:\:\right.(6)$$

The maximum (most significant) change point ($\:{K}_{\tau\:}$) is calculated by:

7a $${K_\tau } = {U_{\tau ,\;T\;}} = \max \left| {{U_{t,\;T\;}}} \right|,1\; \leqslant t < T\;\;$$

Or, the above expression can be rewrite by rank transformation as;

$$\:{K}_{\tau\:}=\begin{array}{c}max\\\:1\:\le\:t<T\end{array}\left|2\sum\:_{i=1}^{t}{R}_{i}-t(T+1)\right|\:\:\:\:\:\:\:\:\:\:\:\:\:\:\:\:\:\:\:\:\:\:\:\:\:\:\:\:\left(7\text{b}\right)$$

Now, to observe the significance change of the test statistic based on the exponential probability distribution is obtained by^47^:

$$\:p\approx\:2\text{n}\text{exp}\left(\frac{-6{K}_{\tau\:}^{2}}{{T}^{3}-{T}^{2}}\right)\:\:\:\:\:\:\left(8\right)$$

Notes: If $\:p<\alpha\:$ (typically 0.05), the null hypothesis ($\:{H}_{0})$ of no change is rejected, indicating a significant change in the dataset.

### PELT test

An innovative technique for finding multiple change points under mild conditions is the PELT method^49^. The technique is precise and, in moderate conditions, has a computing cost that is linear in the amount of data points. Optimal Partitioning^58^algorithm serves as the foundation for this method, which includes a pruning stage in the dynamic programming. The method’s computing cost is decreased by pruning, but the final method’s accuracy is unaffected as it has high scalability and exact accuracy. Both Binary Segmentation^59^and Optimal Partitioning^58^have been evaluated with PELT in simulations. The PELT may be orders of magnitude quicker than Optimal Partitioning (OP), which has exact accuracy but moderate scalability, especially for large data sets, and achieves linear computing cost in these simulations^49^.

In the context of multiple change point detection, a common objective is to minimize a cost function that balances the segmentation of data with a penalty term to avoid over fitting^49^. This is typically expressed as:

$$\:\sum\:_{i=1}^{T+1}\left[C\left({X}_{{\tau\:}_{i-1}+1},\:\dots\:\dots\:\dots\:\dots\:.,\:{X}_{{n}_{i}}\right)\right]+\beta\:f\left(m\right)\:\:\:\:\:\:\:\:\:\:\:\:\:\:\left(9\right)$$

Where:

- $\:C\left({X}_{{\tau\:}_{i-1}+1},\:\dots\:\dots\:\dots\:\dots\:.,\:{X}_{{\tau\:}_{i}}\right)$ is the cost function for each segment of the data series
- $\:\beta\:f\left(m\right)$ represents the penalty for introduction additional change points
- $\:m$ is the number of change points.

The most commonly used cost function in change point analysis is twice the negative log-likelihood, but other functions such as quadratic loss or cumulative sums are also used. The penalty function typically used in practice is linear with respect to the number of change points, i.e., βf(m)=βm.

A commonly used for the penalty term includes:

- Akaike Information Criterion (AIC); β= (2par), where par is the number of parameters.
- Schwarz Information Criterion (SIC or BIC); β= (par log obs), where obs is the number of observations.

These penalties help balance model complexity with goodness of fit, and the PELT method is designed to optimize these linear cost functions efficiently. For small datasets, AIC is typically more reliable as BIC penalizes complexity more aggressively.

The PELT method is a dynamic programming approach to change point detection that improves upon the OP method by introducing pruning. This method minimizes the cost function:

10 $$\:\sum\:_{i=1}^{T+1}\left[C\left({X}_{{\tau\:}_{i-1}+1},\:\dots\:\dots\:\dots\:\dots\:.,\:{X}_{{\tau\:}_{i}}\right)\right]+\beta$$

Where; β is the penalty term. This is equivalent to the earlier formulation in equation (Eq. 10), where the penalty function f(m) = m is linear in the number of change points.

To eliminate the unnecessary change points from each step, the recursive structure of the PELT algorithm can be described as bellow:

- Initial Segmentation: Start by calculating the optimal segmentation for the first data point (K_1,_ say first turn), and proceed recursively.
- Recursive Minimization: For each change point n, minimize the cost function up to that point:

$$\:{K}_{\tau\:}=\begin{array}{c}min\\\:j<K\end{array}\left(K\left(j\right)+\left[C\left({X}_{{\tau\:}_{i-1}+1},\:\dots\:\dots\:\dots\:\dots\:.,\:{X}_{{n}_{i}}\right)\right]+\beta\:\right)\:\:\:\:\:\:\:\:\:\:\:\:\:\:\:\:\:\:\:\:\:\:\:\:\:\:\:\:\left(11\right)$$

The pruning process ensures that for every iteration, only valid change point candidates are considered, effectively reducing the number of computations. When calculating K(n) for a point n, the minimization process covers all previous change points, i.e., j = 0, 1…,*n*− 1. However, pruning eliminates invalid candidates to reduce the search space. In this study, the novel PELT methodology approach by many researchers^49,60,61,62^ and we have employed for the analysis of SPI-1 and SPI-3 time scales to observe the significant multiple changes in the monthly time series.

### Innovative polygon trend analysis

IPTA was used in the identification of trends in rainfall, and it was observed that it offered a few benefits over other conventional trend approaches^43^. The IPTA provides categorisation and visual interception of datasets with uniform length and slope throughout two successive periods without requiring the use of any specific presumptions. Subsequent to its widespread recognition, the IPTA was also examined using rainfall data over different time periods^46,63^.

Following all of these excellent, cutting-edge trend studies, we have used monthly SPI values in the IPTA to support the meteorological drought analysis. The monthly SPI (SPI-1 & SPI-3) was split into two halves (parts) of each month (January, February,…, December) by considering the parameters of standard deviation and mean. Each month’s size (length) and trend slope are determined as follows:

Length (size between each month) say AB is given by;

12 $$\:\:\left|AB\right|=\sqrt{{\left({x}_{2}-{x}_{1}\right)}^{2}+{\left({y}_{2}-{y}_{1}\right)}^{2}}$$

Trend Slope (s).

13 $$\:s=\frac{{y}_{2}-{x}_{2}}{{y}_{1}-{x}_{1}}$$

Where, $\:{x}_{1}$ and $\:{x}_{2}$ are two consecutive periods in first half in x-axis and $\:{y}_{1}$ and $\:{y}_{2}$are two consecutive periods in second half in y-axis respectively. Then the graph are drawn in Cartesian coordinates system of 1:1 (45º) representing increasing trend in above triangle and decreasing trend in lower triangle^36^.

### Long-term trend analysis of drought using scaling hypothesis (Hurst Coefficient)

Three hypothetical tests make up the scaling hypothesis of the Modified Mann Kendall test: the OM (Original Mann-Kendall trend test), HP (Hurst Parameters test), and LT (Mann Kendall long-term persistence, or Mann Kendall LTP). For each test, Hamed (2008)^64^gave the mathematical framework and background, elucidating that $\:{H}_{0n}$ is the null hypothesis and H_1n_is the alternative hypothesis; in the process, n stands for OM, HP, and LT. The process reveals that, Hurst estimator or Hurst Coefficient, He > 0.5 is long term persistence (LTP), and He < 0.5 is anti-persistent (AP) or short- term persistence^31,65^. The identification of the trend in scaling hypothesis is based on the probability function (p-value). $\:{H}_{0}$ (no trend) is acceptable if the p-value is greater than 0.05, while H_1_(there is a trend) is correct if the p-value is 0.05 or below. More substantial trends arise when the probability value is smaller. While conducting this trend test, HK-process package was used in R-software^66^. The Hurst Coefficient (He) in HK-process can be estimated by;

14 $$\:{\sigma\:}^{k}={k}^{He-1}\sigma$$

Where $\:\sigma\:\:$ (standard deviation), k is the time scales and *He* is the entropy production in logarithmic time (ranging 0 to 1).

Procedure of trend identification is given below^31,65^.

a) Presence of **H**_**0**_ will always be considered as no trend exist,

b) Presence of **H**_**1**_ will always be considered as significant trend exist,

c) Presence of **H**_**0OM**_ define no significant trend **(H**_**0**_**)** in Original Mann Kendall test (OM) (End here),

d) If presence of **H**_**1OM**_, define significant trend exist (**H**_**1**_**)** in Original Mann Kendall test (OM) then move to step (e),

e) Presence of **H**_**0HP**_ define no significant trend in Hurst parameter test (HP) (End here),

f) If presence of **H**_**1HP**_, define significant trend exist in Mann Kendall LTP then move to step (g),

g) Presence of **H**_**0LT**,_ define no significant trend exist (End here),

h) If presence of **H**_**1LT**,_ define significant trend exist under LTP.

Given the monthly time frame of the analysis, we have included all three steps of the modified Mann-Kendall test, even in the absence/presence of a significant trend in the step (c) (OM). This deliberate approach will enhance the depth of our analysis by systematically assessing the role of LTP and its potential influence on trend detection. By fully implementing the framework, could provide a more details and precise interpretation of the data, ensuring a more rigorous and insightful assessment of drought variability.

## Results

### Standardized precipitation index

**Fig. 3:**
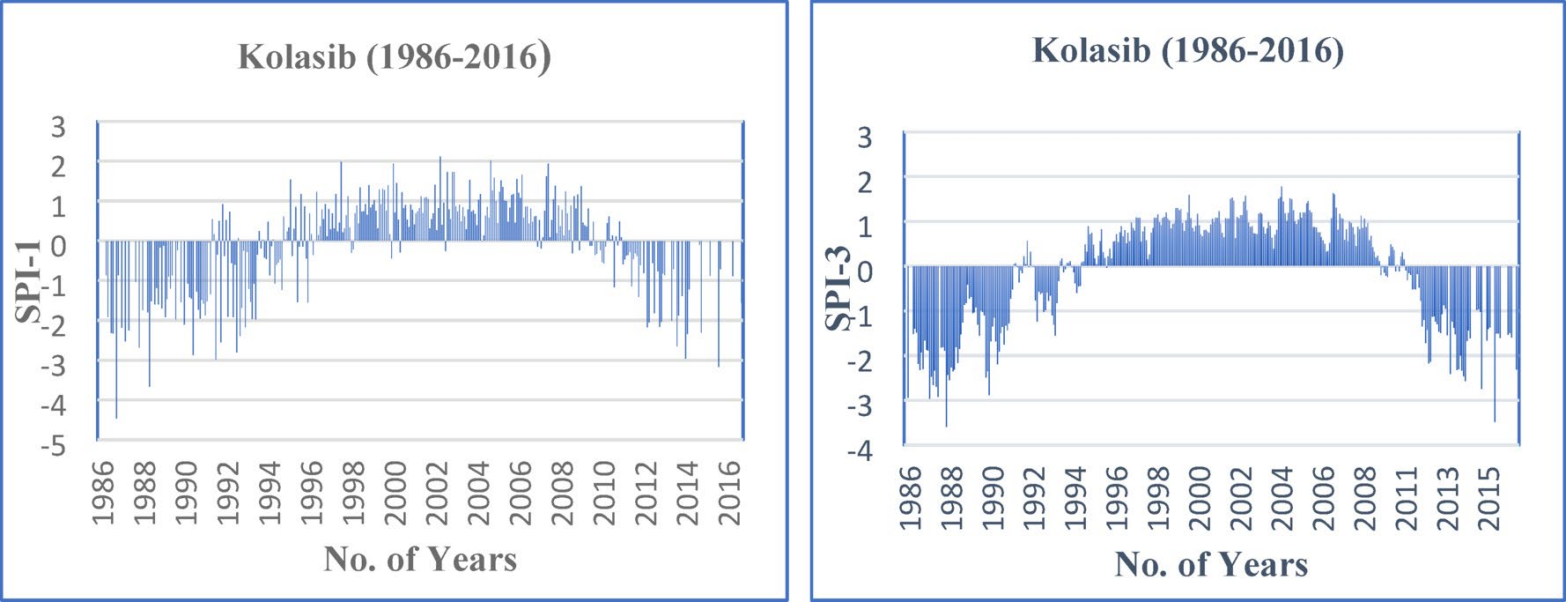
Time series plot of SPI-1 and SPI-3.

The temporal distribution of the SPI values for one and three month time series of Kolasib (Mizoram) are presented in Fig. 3. We have observed from the time series plot of SPI-1 that the Kolasib has been experiencing drought after the year 1986 and drought situation continued for several years (1986–1995). Results of SPI-1 also revealed that the site was in a wetness regime from the year 1996 to 2008 and thereafter, this site experienced a continued drought phenomenon from 2009 onwards. Similarly from the time series plot of SPI-3, it was observed that initial drought period started and was visible from the year 1986 and continued till the year 1992 with minor fluctuations in between during the period 1986–1992. From SPI-3 plots, it can also be seen that the selected site experienced wet period from the year 1996 onwards till 2009. Similar trends of continuous drought regime were observed from the year 2011 onwards at Kolasib. The maximum drought events in monthly time scales (SPI-1 and SPI-3) are also presented in Table 2. The characterization of drought into moderate, severe and extreme was done on the basis of Sharma et al.^64^. In case of the SPI-1, the highest (second highest) cases of ‘moderate’ drought events, i.e., four (three) numbers were observed in the month of July (May and November). Similarly the highest (three) ‘severe’ drought events were observed in the months of February, April, August, November and December. Likewise, the highest (second highest) cases of ‘extreme’ drought events, i.e., four (three) numbers were observed in the month of December (March, July, September and October) at Kolasib.

**Table 2:** Maximum drought event in monthly time series (SPI-1 & SPI-3).

Time Series	Drought Characteristics					
	Moderately dry	Severely dry	Extremely dry	Moderately dry	Severely dry	Extremely dry
	SPI-1			SPI-3		
January	2	2	2	4	5	0
February	0	3	1	3	1	4
March	0	2	3	2	1	4
April	1	3	1	1	1	4
May	3	1	2	1	4	2
June	2	2	1	5	2	3
July	4	2	3	6	1	4
August	1	3	1	3	4	1
September	0	1	3	4	0	4
October	2	2	3	1	4	2
November	3	3	1	2	4	2
December	1	3	4	4	2	3

While in case of the SPI-3 month analysis, the highest six (five followed by four) cases of ‘moderate’ drought events were observed in the month of July, i.e., Monsoon (June, and in the months of January, September and December) in Kolasib. Also, the highest (second highest) cases of ‘severe’ drought events, i.e., five (four) numbers were observed in January (in the months of May, August, October and November). Similarly the highest (four) ‘extreme’ drought events were observed in the months of February, March, April, July and September. The extremely dry case event is seen mostly in the period of pre-monsoon and monsoon season. It was observed that in all the drought classifications for SPI-3, the monsoon season reflects the maximum drought events in Kolasib.

### Estimation of maximum drought attributes- severity, duration and interval

The effects of time scale on drought characteristics, particularly drought severity, duration and interval was analysed and presented in Table 3. Table 3 revealed that for the SPI-1 month (SPI-3 month), the maximum values of drought severity, drought duration and interval were found to be −11.38, 7 months from October, 1990 to April, 1991, and 176-months from March, 1996 to October, 2010 (−34.56 and 17-months from December, 1989 to April, 1991, and 222-months from September, 1993 to February, 2012), respectively over the selected site from the NE region. It is worthwhile to mention that drought severity (duration) increased three times (almost 2.5 times) through the SPI-3 in comparison to the SPI-1 at Kolasib.

**Table 3:** Maximum drought severity, duration, and interval occurrence.

Drought Attributes	SPI-1	SPI-3
Drought Severity	−11.38 (October, 1990 - April, 1991)	−34.56 (June, 1986 –September, 1987)
Drought Duration (month)	7 (October, 1990 -April, 1991)	17 (December, 1989 –April, 1991)
Drought Interval (month)	176 (March, 1996 – October, 2010)	222 (September, 1993 – February, 2012)

**Fig. 4:**
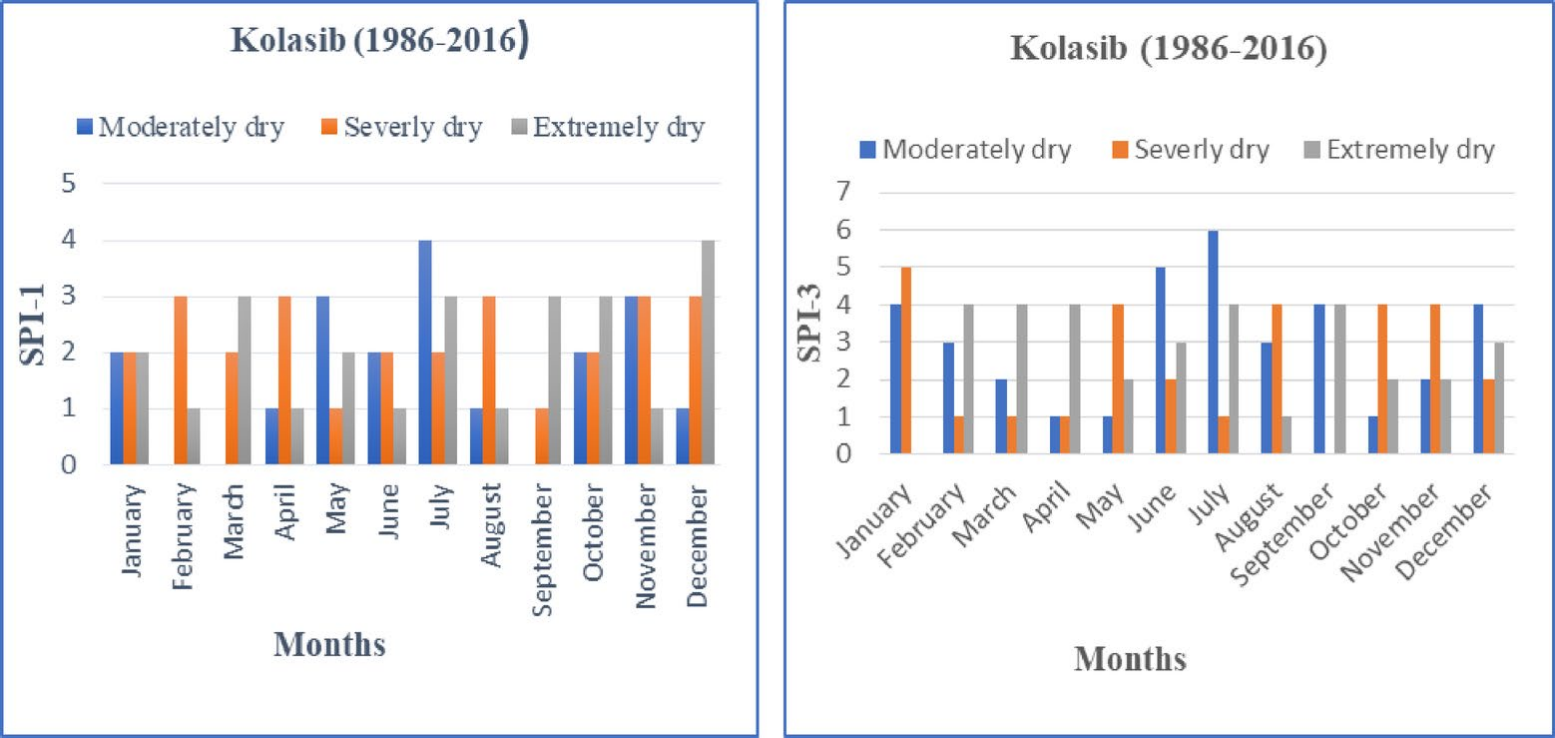
Graph showed the characteristic of Drought events for SPI-1 and SPI-3.

**Fig. 5:**
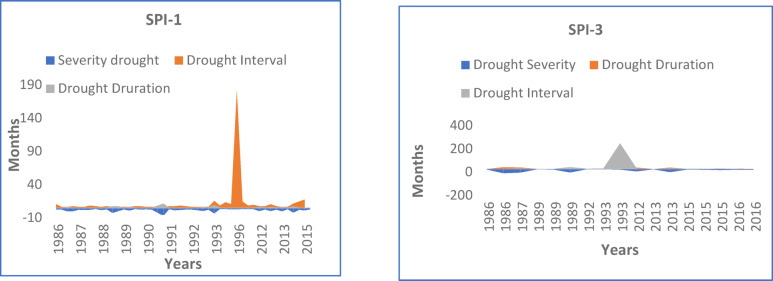
Plot of drought attributes for SPI-1 and SPI-3.

The results show an increase in drought severity from SPI-1 to SPI-3. Similar results were also reported^67,68^ for increase in drought severity with the increase in SPI time scales in the Bundelkhand region of Madya Pradesh (India). Graphical representations of drought events (SPI-1 and SPI-3) are shown in Figs. 4 and 5. These results interpret that although the chance of drought occurring in the case of SPI-1 is relatively low, the longer time scales (more than SPI-1) has a higher chance of detecting a drought event. This is because the time series exhibits notable fluctuations or unpredictability due to the 1-month time frame^69^.

### Change point detection

The CPD analysis for SPI-1 and SPI-3 data set was carried out using the Pettitt test for identifying single maximum change detection and the PELT test for identifying multiple significant change points over the entire time series. The findings from the Pettitt and PELT test provided the valuable insights into drought and wetness trends over time.

**Fig. 6:**
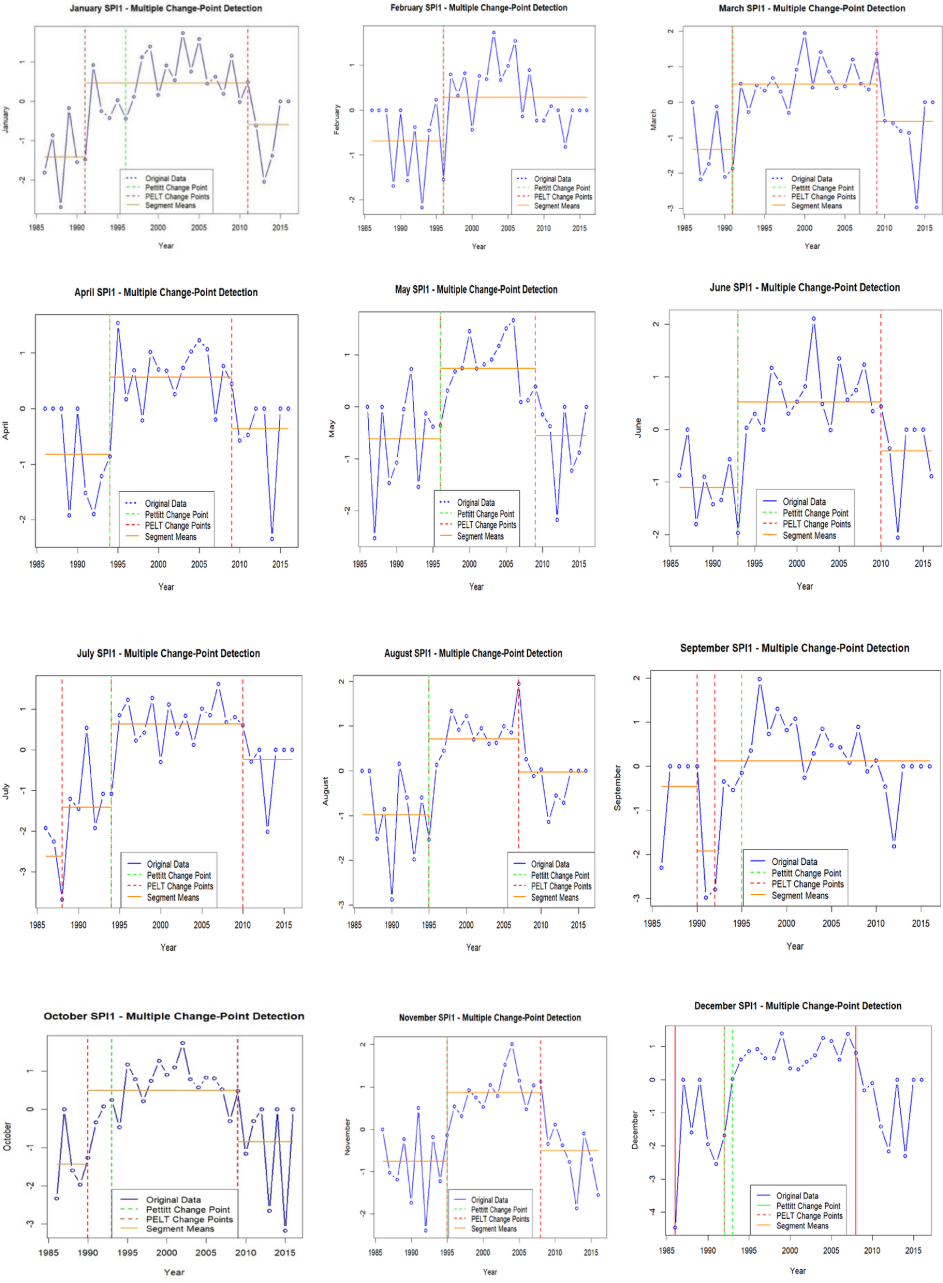
Graphical representation of monthly SPI-1 of most single and multiple CPD by Pettitt and PELT test.

From the Table 4; Fig. 6 for SPI-1, the Pettitt test reflected the significant shifts indicating transitions between dry and wet periods. For instance, for the month of January 1996, a change point was detected with a change value of −0.44 (*p* = 0.03), signifying a shift in climatic conditions. Other months follow the same transition from dry spell to wet spell except for the month of August where transition from wet to dry spell was recorded. Moreover, it was the first maximum turn identified by the Pettitt test. To capture and identify more significance changes, the PELT test further identified multiple change points, such as in 1991 and 2011, dividing the data into three segments with mean values of −1.4233 (1986–1991), 0.4548 (1991–2011), and − 0.5900 (2011–2016). The statistical significance between these segments (*p* = 0.0015 for S1 vs. S2 and *p* = 0.0440 for S2 vs. S3) indicates that significant climatic shifts occurred, influencing drought and wetness cycles on an average of 5 (dry) to 20 (wet) years. Other months exhibit similar patterns, except February month of the year 1996 showing a shift from − 0.6891 (1986–1996) to 0.2824 (1996–2016), with *p* = 0.0059, and March 1991) and September 1992 (showing a shift from − 0.6891 (1986–1996) to 0.2824 (1996–2016), with *p* = 0.0059, and March 1991) indicating notable drought-to-wet-to transitions.

**Table 4:** Monthly change point detection (Pettitt and PELT test) of SPI-1 time scale.

Months	Pettit Test			Multiple change point detection (PELT Test)			
	Change Point, CP (year)	Change value	*P*-value	Change point (year)	Segments (S)	Mean Value of CP	*P*-value
SPI-1							
Jan	1996	−0.44	0.03	1991 & 2011	$$\:\left\{\begin{array}{c}S1=1986-1991\\\:S2=1991-2011\\\:S3=2011-2016\end{array}\right.$$	$$\:\left\{\begin{array}{c}-1.4233\\\:0.4548\\\:-0.5900\end{array}\right.$$	$$\:\left\{\begin{array}{c}S1\:vs\:S2;p=0.0015\\\:S2\:vs\:S3;p=\:0.0440\end{array}\right.$$
Feb	1996	−1.55	0.05	1996	$$\:\left\{\begin{array}{c}S1=1986-1996\\\:S2=1996-2016\end{array}\right.$$	$$\:\left\{\begin{array}{c}-0.6891\\\:0.2824\end{array}\right.$$	$\:S1\:vs\:S2;p=0.0059$
Mar	1991	−1.87	0.17	1991 & 2009	$$\:\left\{\begin{array}{c}S1=1986-1991\\\:S2=1991-2009\\\:S3=2009-2016\end{array}\right.$$	$$\:\left\{\begin{array}{c}-1.3350\\\:0.5079\\\:-0.5487\end{array}\right.$$	$$\:\left\{\begin{array}{c}S1\:vs\:S2;p=0.0043\\\:S2\:vs\:S3;p=\:0.0475\end{array}\right.$$
Apr	1994	−0.86	0.07	1994 & 2009	$$\:\left\{\begin{array}{c}S1=1986-1994\\\:S2=1994-2009\\\:S3=2009-2016\end{array}\right.$$	$$\:\left\{\begin{array}{c}-0.8233\\\:0.5681\\\:-0.3662\end{array}\right.$$	$$\:\left\{\begin{array}{c}S1\:vs\:S2;p=0.0010\\\:S2\:vs\:S3;p=\:0.0687\end{array}\right.$$
May	1996	−0.35	0.07	1996 &2009	$$\:\left\{\begin{array}{c}S1=1986-1996\\\:S2=1996-2009\\\:S3=2009-2016\end{array}\right.$$	$$\:\left\{\begin{array}{c}-0.6136\\\:\:0.7329\\\:-0.5487\end{array}\right.$$	$$\:\left\{\begin{array}{c}S1\:vs\:S2;p=0.0007\\\:S2\:vs\:S3;p=\:0.0027\end{array}\right.$$
Jun	1993	−1.21	0.02	1993 & 2010	$$\:\left\{\begin{array}{c}S1=1986-1993\\\:S2=1993-2010\\\:S3=2010-2016\end{array}\right.$$	$$\:\left\{\begin{array}{c}-1.1062\\\:0.5206\\\:-0.4071\end{array}\right.$$	$$\:\left\{\begin{array}{c}S1\:vs\:S2;p=0.0000\\\:S2\:vs\:S3;p=0.0291\end{array}\right.$$
Jul	1994	−1.08	0.01	1988, 1994 & 2010	$$\:\left\{\begin{array}{c}S1=1986-1988\\\:S2=1988-1994\\\:\genfrac{}{}{0pt}{}{S3=1994-2010}{S4=2010-2016}\end{array}\right.$$	$$\:\left\{\begin{array}{c}-2.6133\\\:-1.4086\\\:\genfrac{}{}{0pt}{}{0.6306}{-0.2400}\end{array}\right.$$	$$\:\left\{\begin{array}{c}S1\:vs\:S2;p=0.1504\\\:S2\:vs\:S3;p=0.0042\\\:S3\:vs\:S4;p=0.0336\end{array}\right.$$
Aug	1995	1.54	0.01	1995 & 2007	$$\:\left\{\begin{array}{c}S1=1986-1995\\\:S2=1995-2007\\\:S3=2007-2016\end{array}\right.$$	$$\:\left\{\begin{array}{c}-0.9800\\\:0.7085\\\:-0.0290\end{array}\right.$$	$$\:\left\{\begin{array}{c}S1\:vs\:S2;p=0.0004\\\:S2\:vs\:S3;p=0.0433\end{array}\right.$$
Sep	1995	−0.15	0.02	1990 & 1992	$$\:\left\{\begin{array}{c}S1=1986-1990\\\:S2=1990-1992\\\:S3=1992-2016\end{array}\right.$$	$$\:\left\{\begin{array}{c}-0.4620\\\:-1.9267\\\:0.1176\end{array}\right.$$	$$\:\left\{\begin{array}{c}S1\:vs\:S2;p=0.2660\\\:S2\:vs\:S3;p=0.1637\end{array}\right.$$
Oct	1994	−0.47	0.10	1990 & 2009	$$\:\left\{\begin{array}{c}S1=1986-1990\\\:S2=1990-2009\\\:S3=2009-2016\end{array}\right.$$	$$\:\left\{\begin{array}{c}-1.4340\\\:0.4910\\\:-0.8512\end{array}\right.$$	$$\:\left\{\begin{array}{c}S1\:vs\:S2;p=0.0057\\\:S2\:vs\:S3;p=0.0275\end{array}\right.$$
Nov	1995	−0.14	0.09	1995 & 2008	$$\:\left\{\begin{array}{c}S1=1986-1995\\\:S2=1995-2008\\\:S3=2008-2016\end{array}\right.$$	$$\:\left\{\begin{array}{c}-0.7630\\\:0.8621\\\:-0.5000\end{array}\right.$$	$$\:\left\{\begin{array}{c}S1\:vs\:S2;p=0.0002\\\:S2\:vs\:S3;p=0.0014\end{array}\right.$$
Dec	1992 & 1993	−1.68 & 0.02	0.07	1992 & 2008	$$\:\left\{\begin{array}{c}S1=1986-1992\\\:S2=1992-2008\\\:S3=2008-2016\end{array}\right.$$	$$\:\left\{\begin{array}{c}-1.7471\\\:0.6212\\\:-0.6100\end{array}\right.$$	$$\:\left\{\begin{array}{c}S1\:vs\:S2;p=0.0057\\\:S2\:vs\:S3;p=0.0097\end{array}\right.$$

**Fig. 7:**
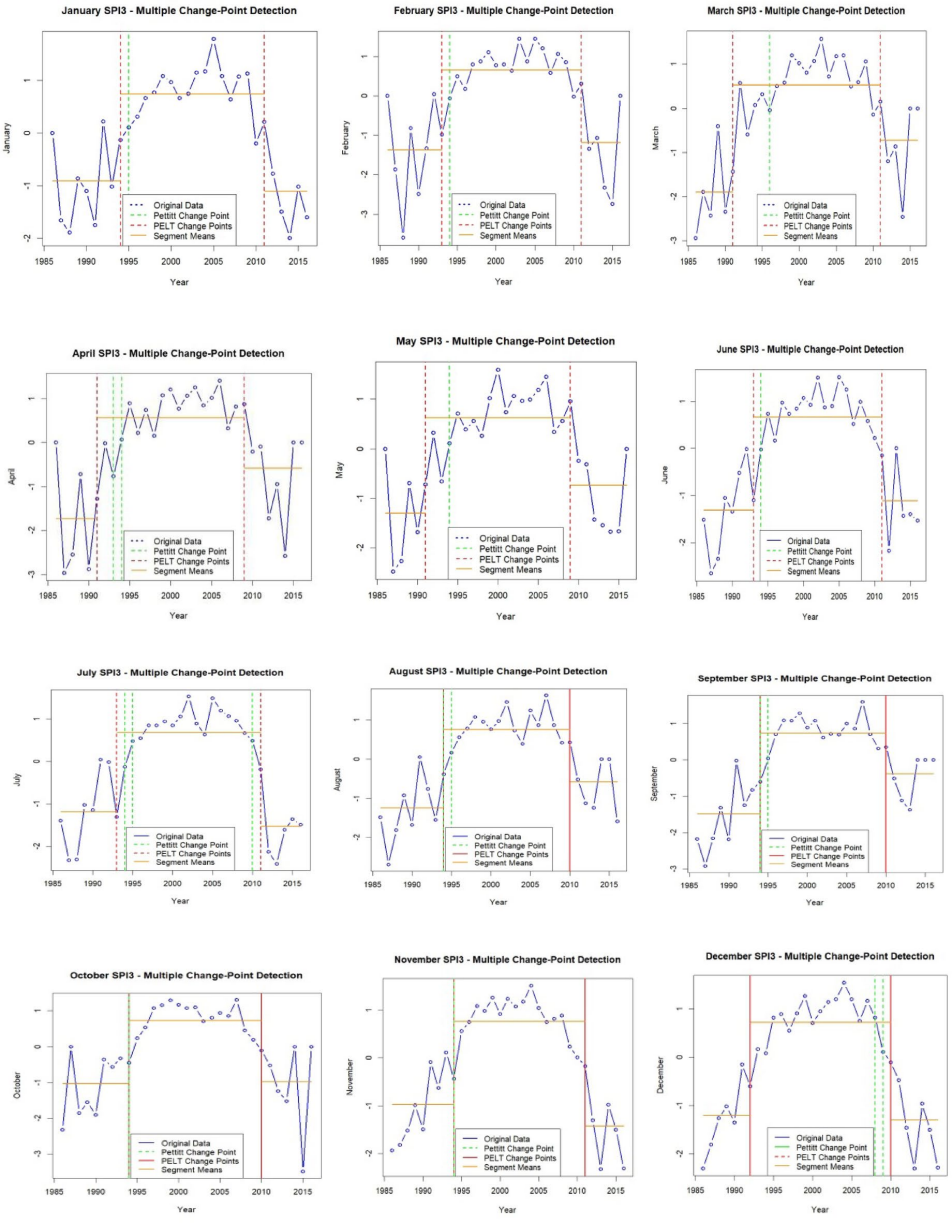
Graphical representation of monthly SPI3 of most single and multiple CPD by Pettitt and PELT test.

For SPI-3, the results showed the extended dry and wet phases over larger timescales (Table 5; Fig. 7). The Pettitt test identifies changes such as for the month of January 1995 (change value = 0.10, *p* = 0.063) and February 1994 (change value = −0.06, *p* = 0.046), pointing to shifts in long-term climate patterns. The PELT test further detects multiple change points, such as 1994 and 2011 for January, with mean SPI values transitioning from − 0.9100 (1986–1994) to 0.7317 (1994–2011) and then declining to −1.1100 (2011–2016). The statistical significance of these transitions (*p* = 0.0001 for S1 vs. S2 and *p* = 0.0012 for S2 vs. S3) confirms notable climate variability. February also follows a similar pattern, with shifts from − 1.3800 (1986–1993) to 0.6505 (1993–2011) and then to −1.1950 (2011–2016), reflecting alternating dry and wet phases. It is noteworthy to mention that by using PELT test for larger time scale (SPI-3), we have identified a notably significance continuous drought in the following months of November (mean= −1.43), December (−1.3), January (−1.11), and June (−1.113), July (−1.532, highest drought effect), August (−0.579), respectively.

**Table 5:** Monthly change point detection (Pettitt and PELT test) of SPI-3 time scale.

Months	Pettit Test			Multiple change point detection (PELT Test)			
	Change Point, CP (year)	Change value	*P*-value	Change point (year)	Segments (S)	Mean Value of CP	*P*-value
SPI-3							
Jan	1995	0.1	0.063	1994 & 2011	$$\:\left\{\begin{array}{c}S1=1986-1994\\\:S2=1994-2011\\\:S3=2011-2016\end{array}\right.$$	$$\:\left\{\begin{array}{c}-0.9100\\\:0.7317\\\:-1.1100\end{array}\right.$$	$$\:\left\{\begin{array}{c}S1\:vs\:S2;p=0.0001\\\:S2\:vs\:S3;p=0.0012\end{array}\right.$$
Feb	1994	−0.06	0.046	1993 & 2011	$$\:\left\{\begin{array}{c}S1=1986-1993\\\:S2=1993-2011\\\:S3=2011-2016\end{array}\right.$$	$$\:\left\{\begin{array}{c}-1.3800\\\:0.6505\\\:-1.1950\end{array}\right.$$	$$\:\left\{\begin{array}{c}S1\:vs\:S2;p=0.0020\\\:S2\:vs\:S3;p=0.0124\end{array}\right.$$
Mar	1996	−0.04	0.028	1991 & 2011	$$\:\left\{\begin{array}{c}S1=1986-1991\\\:S2=1993-2011\\\:S3=2011-2016\end{array}\right.$$	$$\:\left\{\begin{array}{c}-1.9083\\\:0.5200\\\:-0.7300\end{array}\right.$$	$$\:\left\{\begin{array}{c}S1\:vs\:S2;p=0.0005\\\:S2\:vs\:S3;p=0.0270\end{array}\right.$$
Apr	1993 1994	−0.77 0.07	0.035	1991 & 2009	$$\:\left\{\begin{array}{c}S1=1986-1991\\\:S2=1991-2009\\\:S3=2009-2016\end{array}\right.$$	$$\:\left\{\begin{array}{c}-1.7317\\\:0.5584\\\:-0.5837\end{array}\right.$$	$$\:\left\{\begin{array}{c}S1\:vs\:S2;p=0.0050\\\:S2\:vs\:S3;p=0.0232\end{array}\right.$$
May	1994	0.11	0.070	1991 & 2009	$$\:\left\{\begin{array}{c}S1=1986-1991\\\:S2=1991-2009\\\:S3=2009-2016\end{array}\right.$$	$$\:\left\{\begin{array}{c}-1.3033\\\:0.6232\\\:-0.7350\end{array}\right.$$	$$\:\left\{\begin{array}{c}S1\:vs\:S2;p=0.0036\\\:S2\:vs\:S3;p=0.0050\end{array}\right.$$
Jun	1994	−0.02	0.039	1993 & 2011	$$\:\left\{\begin{array}{c}S1=1986-1993\\\:S2=1993-2011\\\:S3=2011-2016\end{array}\right.$$	$$\:\left\{\begin{array}{c}-1.3162\\\:0.6595\\\:-1.1133\end{array}\right.$$	$$\:\left\{\begin{array}{c}S1\:vs\:S2;p=0.0002\\\:S2\:vs\:S3;p=0.0024\end{array}\right.$$
Jul	1994 & 2010	−0.13 & 0.49	0.120	1993 & 2011	$$\:\left\{\begin{array}{c}S1=1986-1993\\\:S2=1993-2011\\\:S3=2011-2016\end{array}\right.$$	$$\:\left\{\begin{array}{c}-1.1875\\\:0.6779\\\:-1.5317\end{array}\right.$$	$$\:\left\{\begin{array}{c}S1\:vs\:S2;p=0.0003\\\:S2\:vs\:S3;p=0.0003\end{array}\right.$$
Aug	1994 1995	−0.38 & 0.17	0.012	1994 & 2010	$$\:\left\{\begin{array}{c}S1=1986-1994\\\:S2=1994-2010\\\:S3=2010-2016\end{array}\right.$$	$$\:\left\{\begin{array}{c}-1.2456\\\:0.7635\\\:-0.5786\end{array}\right.$$	$$\:\left\{\begin{array}{c}S1\:vs\:S2;p=0.000\\\:S2\:vs\:S3;p=0.0024\end{array}\right.$$
Sep	1994 & 1995	−0.60 & 0.04	0.004	1994 & 2010	$$\:\left\{\begin{array}{c}S1=1986-1994\\\:S2=1994-2010\\\:S3=2010-2016\end{array}\right.$$	$$\:\left\{\begin{array}{c}-1.4933\\\:0.7347\\\:-0.3771\end{array}\right.$$	$$\:\left\{\begin{array}{c}S1\:vs\:S2;p=0.000\\\:S2\:vs\:S3;p=0.0028\end{array}\right.$$
Oct	1994	−0.45	0.028	1994 & 2010	$$\:\left\{\begin{array}{c}S1=1986-1994\\\:S2=1994-2010\\\:S3=2010-2016\end{array}\right.$$	$$\:\left\{\begin{array}{c}-1.0378\\\:0.7265\\\:-0.9814\end{array}\right.$$	$$\:\left\{\begin{array}{c}S1\:vs\:S2;p=0.0001\\\:S2\:vs\:S3;p=0.0107\end{array}\right.$$
Nov	1994	−0.44	0.090	1994 & 2011	$$\:\left\{\begin{array}{c}S1=1986-1994\\\:S2=1994-2011\\\:S3=2011-2016\end{array}\right.$$	$$\:\left\{\begin{array}{c}-0.9778\\\:0.7561\\\:-1.4300\end{array}\right.$$	$$\:\left\{\begin{array}{c}S1\:vs\:S2;p=0.0000\\\:S2\:vs\:S3;p=0.0007\end{array}\right.$$
Dec	2009	0.11	0.115	1992 & 2010	$$\:\left\{\begin{array}{c}S1=1986-1992\\\:S2=1992-2010\\\:S3=2010-2016\end{array}\right.$$	$$\:\left\{\begin{array}{c}-1.2129\\\:0.7142\\\:-1.3000\end{array}\right.$$	$$\:\left\{\begin{array}{c}S1\:vs\:S2;p=0.0001\\\:S2\:vs\:S3;p=0.0004\end{array}\right.$$

### Innovative polygon trend analysis (IPTA)

**Fig. 8:**
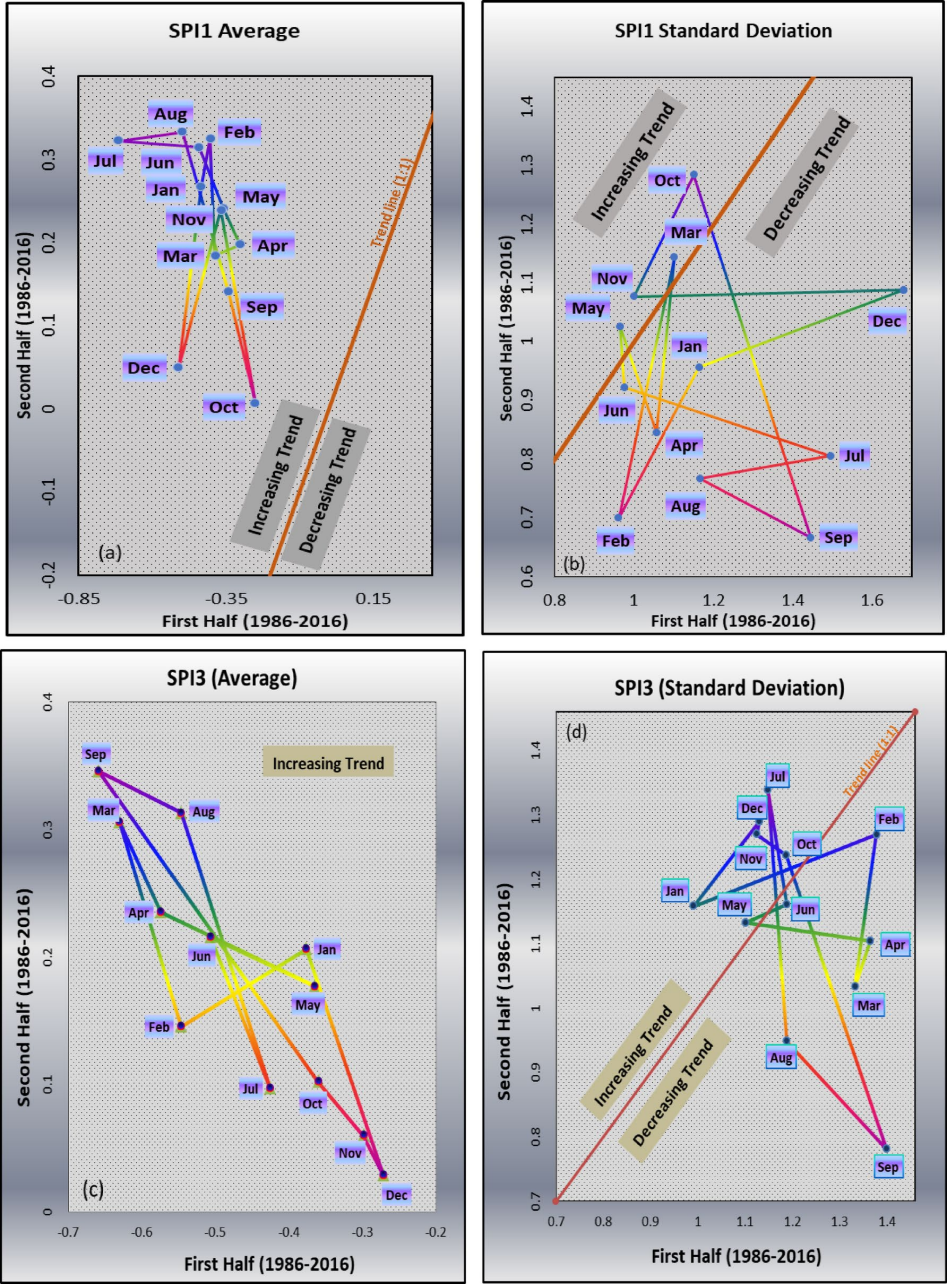
Graphical Representation of Innovative Polygon Trend for both SPI-1 & SPI-3 with Parameters Average [(**a**). SPI-1 & (**c**). SPI-3)] and Standard Deviation [(**b**). SPI-1 & (**c**). SPI-3].

The IPTA’s graphical representations for both the SPI-1 and the SPI-3 are shown in Fig. 8. The SPI-1 arithmetic average (Fig. 8a) of IPTA graphs show strong increasing trends for all the months above the 1:1 line. The highest trend slope (−8.522) indicated the highest rate of change was observed from February to March, which further indicates the transition between these two months with trend length of 0.141 respectively (Table 6). The trend line is drawn as Cartesian Co-ordinate System (CCS) of 1:1 (at 45º) indicating upper triangle as increasing trend and lower triangle as decreasing trend^36^ and ones falling on the Cartesian line indicate no transition (change). The increasing trend over the analyzed period revealed, that there is a tendency for the values to rise, suggesting a potential increase in the parameter being studied, i.e. precipitation in this case. The standard deviation graph of SPI-1 (Fig. 8b) showed certain transition in each month notably March, May, October and November as increasing trends, whereas, for the remaining months (Jan, Feb, Apr, Jun, Jul, Aug, Sep, and Dec) as decreasing trends, where, fall below Cartesian trend line (1:1). From Table 6, it was also observed that June month depicts the largest decline from May at a transition of trend slope (−9.455) and trend length (0.105), while March month depicts the highest increase from February at a transition of trend slope (6.75). Similarly, for the SPI-3 arithmetic average plot (Fig. 8c) all months fall in upper triangle indicating increasing trend. From Table 6 (SPI-3), the highest transition trend slope (1.788) was observed between July to August month having trend length (0.247). The SPI-3 standard deviation plot (Fig. 8d) from the mean for the months of January, May, July, October, November, and December falls in the upper triangle (above the trend line), suggesting an upward trend. The strongest transition upward trend was seen from November to December. Similarly, the months of February, March, April, June, August, and September fall within the bottom triangle and showed a marked downward tendency. While the largest declining transition trends slopes were observed in July to August (−9.591), and the lowest decreasing trend was observed in February to March (5.203).

**Table 6:** IPTA of SPI-1 and SPI-3 for the parameters of average and standard deviation.

		Jan-Feb	Feb-Mar	Mar-Apr	Apr-May	May-Jun	Jun-Jul	Jul-Aug	Aug-Sep	Sep-Oct	Oct-Nov	Nov-Dec	Dec-Jan	
SPI-1	Average													
	Trend Length	0.066	0.141	0.086	0.072	0.111	0.275	0.219	0.248	0.162	0.260	0.239	0.230	
	Trend Slope	1.754	−8.522	0.152	−0.742	−0.902	−0.030	0.048	−1.224	−1.490	−2.008	1.306	2.867	
	Standard Deviation													
	Trend Length	0.326	0.464	0.300	0.202	0.105	0.531	0.329	0.295	0.683	0.256	0.680	0.531	
	Trend Slope	1.256	3.157	6.750	−1.957	−9.455	−0.224	0.119	−0.360	−2.095	1.355	0.015	0.254	
SPI-3	Average													
	Trend Length	0.181	0.181	0.090	0.217	0.147	0.143	0.247	0.118	0.386	0.075	0.041	0.206	
	Trend Slope	0.360	−1.905	−1.251	−0.278	−0.270	−1.465	−1.788	−0.292	−0.814	−0.689	−1.149	−1.692	
	Standard Deviation													
	Trend Length	0.403	0.241	0.078	0.265	0.092	0.182	0.392	0.269	0.503	0.070	0.021	0.192	
	Trend Slope	0.287	5.203	2.179	−0.109	0.328	−4.322	−9.591	−0.794	−2.149	−0.526	4.350	0.945	

### Estimation of long-term trend analysis of drought under scaling hypothesis (Hurst Coefficient)

The modified Mann-Kendall test under the scaling hypothesis revealed crucial insights into the long-term persistence and monthly drought variability at 1-month (SPI-1) and 3-month (SPI-3) time scales as presented in Table 7. For SPI-1, the Hurst exponent (He) is consistently greater than 0.5 across all months, indicating that drought anomalies exhibit long-term persistence (LTP). The highest persistence is observed in December (He = 0.845) and August (He = 0.838), suggesting a strong memory effects in these months. The Original Mann-Kendall (OM) test did not detect significant trends in any month (all p-values > 0.05), meaning that, under the assumption of independence, no clear increasing or decreasing pattern was evident. However, the Hurst Parameter test (HP) identified the significant LTP in most months (HP ≤ 0.05), except for February (HP = 0.063), where LTP is not significant. When the Mann-Kendall test was adjusted for LTP (LT), no significant trends remained (all LT p-values > 0.05). This indicates that any trends observed under the independence assumption were likely due to memory effects rather than actual climatic shifts.

**Table 7:** Estimation of long-term persistence (LTP) of drought in trend analysis under scaling hypothesis of SPI-1 and SPI-3.

Time series	Hurst estimate, (He)	Original Mann Kendall, *p*-value (OM)	Significance of He, *p*-value (HP)	Mann Kendall, LTP *p*-value (LT)	Trend’s Identification
SPI-1 month					**{H0**_**OM**_**}** No significant trend exists under LTP
**Jan**	0.783	0.148	0.002*	0.631	
**Feb**	0.620	0.240	0.063	0.571	
**Mar**	0.771	0.708	0.003*	0.898	
**Apr**	0.711	0.434	0.011*	0.760	
**May**	0.775	0.395	0.003*	0.773	
**Jun**	0.786	0.196	0.002*	0.669	
**Jul**	0.753	0.106	0.004*	0.566	
**Aug**	0.838	0.332	0.000*	0.775	
**Sep**	0.786	0.496	0.002*	0.822	
**Oct**	0.816	0.865	0.001*	0.958	
**Nov**	0.800	0.563	0.001*	0.853	
**Dec**	0.845	0.552	0.000*	0.862	
SPI-3 month					
**Jan**	0.947	0.255	0.000*	0.789	
**Feb**	0.890	0.269	0.000*	0.770	
**Mar**	0.872	0.103	0.000*	0.654	
**Apr**	0.826	0.227	0.001*	0.715	
**May**	0.887	0.377	0.000*	0.814	
**Jun**	0.906	0.228	0.000*	0.757	
**Jul**	0.965	0.284	0.000*	0.809	
**Aug**	0.913	0.135	0.000*	0.706	
**Sep**	0.927	0.122	0.000*	0.705	
**Oct**	0.890	0.497	0.000*	0.857	
**Nov**	0.962	0.671	0.000*	0.923	
**Dec**	0.968	0.552	0.000*	0.894	

Note: * significance in P-value.

For SPI-3, long-term persistence was even stronger than in SPI-1, as indicated by the higher Hurst exponent values (He > 0.8 for all months). This suggests that monthly drought variability exhibits even greater memory effects compared to the 1-month scale. The highest persistence was observed in December (He = 0.968), November (He = 0.962), and July (He = 0.965), highlighting the strong dependence on past drought patterns. Despite the strong persistence, the Original Mann-Kendall test (OM) did not detect any significant trends (all p-values > 0.05). Meanwhile, HP values are highly significant (all HP ≤ 0.05), confirming strong LTP in all months. When adjusted for this persistence using the LTP-modified Mann-Kendall test (LT), no month retained a significant trend (all LT p-values > 0.05). Therefore, no month showed significant trend for LTP of drought under scaling hypothesis. The SPI-3 observed strong LTP than SPI-1, this may be due to the seasonal effect and large scale data variability.

## Discussion

The findings of this study highlighted significant drought trends and variability in Kolasib, Mizoram, using the advanced statistical approaches. The SPI analysis revealed that drought intensity increases with longer time scales, as seen in the higher severity of SPI-3 compared to SPI-1. Numerous researches have also noted increased SPI values for extended time scales of 12 and 24 months^68,70,71^and also reported the importance of SPI at different time scale for accurate drought identification and characterization. Drought, being the natural calamity, severely affected the sustainability of agriculture in several parts of the India. Although the North eastern Himalayan states of India receive a high amount of rainfall but several regions have experienced the alternating dry and wet periods, with prolonged droughts occurring in specific decades due to changes in climatic variability. For the study area, the CPD analysis further confirmed these shifts, with the Pettitt test identifying the most significant single breakpoints, while the PELT algorithm provided a more comprehensive assessment by detecting multiple structural changes. The ability of PELT to capture continuous shifts in climate conditions makes it a more reliable tool for understanding long-term drought variability. Compared to other precise techniques like Segment Neighbourhood algorithms, PELT is a quicker way for detecting change points^49,61^. To avoid overfitting, the AIC and BIC are frequently employed. The AIC performed better in avoiding overfitting in this investigation.

The IPTA revealed strong increasing trends in both SPI-1 and SPI-3, indicating that drought conditions are persisting over time. The strongest transitions were observed in the monsoon and winter months, particularly between July and August for SPI-3 and February to March for SPI-1. This suggests that changes in precipitation patterns during critical agricultural and hydrological periods could have severe consequences for the NEI region for meeting the domestic and agricultural water needs. Findings of this research revealed that the new approach of trend analysis is more sensitive. Previous research has also demonstrated that the IPTA can provide more sensitive results and successfully identify patterns identified by the Mann-Kendall test^63,72,73^. Innovative graphical mean provides both visual and numerical in addition to trend-setting success. Additionally, the LTP analysis using the Hurst exponent confirmed that droughts in Kolasib exhibit strong memory effects, meaning past conditions significantly influence future drought patterns. Higher Hurst exponent values suggested that SPI-3 is more persistent than SPI-1. Any observed trends in drought are statistical anomalies rather than actual changes since neither SPI-1 nor SPI-3 exhibit significant trends after controlling for the LTP. The scaling hypothesis is true for both SPI-1 and SPI-3 as persistence eliminates trends (no significance LTP) that were first seen under independence. In contrast to seasonal drought variability (SPI-3), which shows a greater reliance on historical values, short-term drought anomalies (SPI-1) have less memory effects. Despite the observed variations, no significant trends were detected after adjusting for LTP, suggesting that the fluctuations may be part of a natural drought cycle rather than solely due to climate change. The findings supported the idea that drought patterns identified using traditional means may be deceptive if long-term persistence is not considered. It is suggested that drought variability exhibits a scaling characteristic as opposed to random fluctuations by the existence of substantial Hurst dynamics in the SPI-1 and SPI-3. To prevent incorrect attributions of climate change impacts^74^, it is crucial to use long-term statistical procedures when assessing climatic trends. Natural fluctuations may be misinterpreted as trends by standard trend tests, such as the traditional Mann-Kendall test, which presumes data independence^66^. The scaling hypothesis reduces erroneous trend detections by allowing trend estimates while considering LTP.

Therefore, the information observed here is crucial for Kolasib district, Mizoram. After performing multiple statistical tests, we have observed that winter and monsoon are severely prone to drought conditions. Entire region of Kolasib experienced a rainfall deficit as reported by Saha et al.^75^and the lack of rainfall in Bilkhawthlir (also in Kolasib) has caused a major impact on the agricultural pattern in the region. In the NE India, the El Niño-related drought was not as severe. Beyond El Niño, the rising temperatures and increased monsoon variability due to global climate change may contribute to more frequent and severe droughts, particularly in NER^3^.

## Conclusions

This study provides a comprehensive analysis of meteorological drought patterns for Kolasib, Mizoram (India). Drought characterization was performed using the SPI, IPTA, the CPD methods (Pettitt and PELT), and the LTP analysis. Results revealed a consistent increase in drought severity with longer time scales, as SPI-3 indicated more severe droughts compared to SPI-1, which might be due to seasonal effect. The findings suggested that Kolasib experienced alternating dry and wet periods, with significant droughts observed over extended time frames, particularly in the winter and monsoon seasons. The CPD using both Pettitt and PELT tests provided valuable insights into the transitions between dry and wet periods. The Pettitt test identified major single change points, while the PELT method successfully captured multiple significant shifts, offering a more detailed understanding of the drought cycles over time. November to January and June to August showed the most drought condition in PELT method of SPI-3 month.

The IPTA confirmed strong increasing trends in the SPI values, further indicating rise in drought intensity, particularly from February to March in SPI-1 and July to August in SPI-3. Moreover, finding of both CPD and IPTA tests observed highest drought segment in July and August. It is worth to mention that these months are crucial from the perspective of monsoon activities as nearly 34% of total annual rainfall is recorded in these months, and therefore it may cause significant strain on the economy of this region. Drought may also lead to a decline in precious natural availability including forest products to the local indigenous tribal populace, the majority of which is entirely dependent on forest, agriculture and other allied activities. Results emphasized the urgency of addressing the persistent drought trends and the need for more comprehensive drought management strategies. Additionally, the LTP analysis through the Hurst exponent counters the strong long-term effect of drought in the following month, November to January and June to August, as observed by all the methods. Despite no significant trends being identified after adjusting for LTP, the study shows that the region is likely to experience cyclical drought conditions in future, with no immediate reversal of the trend. As highest shifting drought was observed in monsoon season indicating the climate change effect over the region in the most crucial season.

By incorporating the insights gained from this study, policymakers may develop more robust frameworks for drought mitigation, sustainable resource management, and climate adaptation strategies. The study’s results underscore the importance of using advanced statistical methods, such as, IPTA and PELT, to monitor and manage drought conditions. With these tools, the local authorities can better anticipate droughts, minimize their adverse impacts, and ensure the region’s long-term resilience against climate variability.

## Data Availability

The datasets generated and analysed during the current study are not publicly available. But, corresponding author may provide on reasonable request after obtaining prior permission from the data-agency.
